# Prospection deficits in patients with first-episode schizophrenia: a cross-sectional comparative study

**DOI:** 10.1038/s41537-023-00365-w

**Published:** 2023-06-03

**Authors:** Antoinette C. O. Fung, Rui-ting Zhang, Stanley S. L. Yip, Grace K. S. Poon, Chi-Wai Cheng, Tian-xiao Yang, Simon S. Y. Lui, Raymond C. K. Chan

**Affiliations:** 1 Castle Peak Hospital, Hong Kong Special Administrative Region, China; 2 Neuropsychology and Applied Cognitive Neuroscience Laboratory, CAS Key Laboratory of Mental Health, Institute of Psychology, Chinese Academy of Sciences, Beijing, China; 3 Department of Psychology, Hunan Normal University, Chansha, China; 4 Cognition and Human Behaviour Key Laboratory of Hunan Province, Hunan Normal University, Changsha, China; 5 Department of Psychology, University of Chinese Academy of Sciences, Beijing, China; 6 Department of Psychiatry, School of Clinical Medicine, The University of Hong Kong, Hong Kong Special Administrative Region, China

**Keywords:** Biomarkers, Schizophrenia

## Abstract

Prospection refers to the ability to simulate and pre-experience future events. Schizophrenia patients have difficulty in anticipating pleasure in future events, but previous studies examined prospection deficits in chronic schizophrenia patients. This study aimed to investigate prospection deficits in first-episode schizophrenia patients. Thirty first-episode schizophrenia patients and 31 healthy controls completed the Affective Prospection Task, which utilized pictorial cues to involve positive, neutral and negative prospection. Participants’ ratings regarding the phenomenal characteristics of their prospected events were collected, and their prospected narratives were coded using a valid scoring manual. We also assessed intelligence, working memory and logical memory. The results showed, in all participants, valence of the cues significantly influenced participants’ sense of pre-experience, temporal distance, emotion experience, vividness and participation of the prospected events, as well as the richness of sensory details. The two groups did not differ in self-report phenomenal characteristics of their prospected events. For coded characteristics, schizophrenia patients’ prospected narratives were less rich in thought/emotion than controls, even after controlling for intelligence and memory deficits. We extended empirical evidence for prospection deficits from chronic schizophrenia samples to first-episode schizophrenia patients.

## Introduction

Prospection refers to the ability to simulate and pre-experience future events^1^. During prospection, one may predict emotional consequences on the occurrence of the prospected event, a phenomenon termed “affective forecasting”; in-the-moment feeling during affective forecasting could also induce emotions, a phenomenon called “anticipatory affect”^2^. Prospection is closely related to decision-making, planning, anticipatory pleasure, and motivation^1,3,4^. Prospection is a complex function, involving a wide range of regions, including the medial-temporal lobe (MTL) of the default mode network^5^ and the network proposed by Schacter et al.^6^ which comprises the MTL, posterior cingulate, retrosplenial cortex, medial prefrontal cortex, and lateral temporal and parietal regions^6,7^.

According to the constructive episodic simulation hypothesis^6^, prospecting the future involves past episodic memory, and the flexible recombination of elements from past memory and simulated representations. Schizophrenia is associated with deficits in self-initiation and memory, which may contribute to prospection deficits. Moreover, prospection is closely related to affective forecasting and anticipatory affect^2^. Importantly, prospection deficits could result in anticipatory pleasure deficits in schizophrenia^3^. Meta-analytic studies^8,9^ concluded that patients with schizophrenia anticipated less pleasure than healthy people. According to Frost and Strauss^10^’s model, anticipatory pleasure involves several processes, including prospection, anticipatory affect, affective forecasting, and associative learning. Anticipatory pleasure deficits are closely related to formation of negative symptoms in schizophrenia^11,12^, and therefore research on prospection in patients with schizophrenia can unveil putative cognitive mechanisms for negative symptoms.

However, limited research to-date has investigated prospection deficits in schizophrenia patients. To our knowledge, four previous studies have examined the novel construct of prospection, and the results generally supported the existence of prospection deficits in patients with chronic schizophrenia. For instance, D’Argembeau et al.^13^ utilized positive and negative short sentences as cues to induce prospected narratives, and found that schizophrenia patients’ narratives were less specific (i.e., not indicating specified time and place, appearing categorical or general) than those generated by healthy people. In contrast, Raffard et al.^14^ utilized positive and negative pictures as cues, and found that schizophrenia patients’ prospected narratives were less specific, and they self-reported fewer sensory details, less contextual information self- and other-referential information during prospection. On the other hand, Painter and Kring^3^ included neutral phases as cues, and measured schizophrenia patients’ emotions in relation to prospection. They found that schizophrenia patients self-reported less sensory details and emotion involvement during prospection, and their prospected events were less specific. More recently, Yang et al.^15^ utilized positive, neutral and negative pictures as cues, and found that schizophrenia patients self-reported less pre-experiencing and vivid feelings during prospection than healthy people, and their prospected narratives were less rich compared to those of healthy people. Interestingly, the emotion valence of the cues also affected the quality of prospection. For instance, Painter and Kring^3^ demonstrated that both schizophrenia patients and healthy people self-reported less sensory and contextual experiences in negative cued rather than positive/neutral cued prospection. Negative cues also generated prospected narratives with lower clarity in schizophrenia patients. Yang et al.^15^ demonstrated that positive cues promoted higher specificity and richness of the prospected narratives, and better pre-experiencing, vivid and participation feelings than neutral/negative cues during prospection.

Given that all previous studies^3,13–15^ only utilized chronic schizophrenia sample, it remains unclear whether first-episode schizophrenia patients would have prospection deficits, and whether prospection deficits would remain significant after accounting for schizophrenia-associated memory deficits^6^. This study aimed to examine prospection ability in first-episode schizophrenia patients using pictures as cues for prospection^15^. We hypothesized that first-episode schizophrenia patients would have prospection deficits. We also hypothesized that first-episode schizophrenia patients and controls would be subjected to valence effects of the cues to a comparable extent, similar to our earlier findings in patients with chronic schizophrenia^15^.

## Results

Our sample comprised 30 clinically-stable outpatients with first-episode DSM-IV^16^ schizophrenia and 31 healthy individuals. Schizophrenia patients and controls were matched in age and gender ratio (*p*s > 0.05), but schizophrenia patients had lower years of education (*p* < 0.001) than controls (see Table 1). The schizophrenia group was at clinical stabilization with low PANSS positive (mean = 8.03; SD = 2.43), negative (mean = 11.40; SD = 4.99) and general symptoms (mean = 18.47; SD = 3.96), and HAM-D scores (mean = 0.30; SD = 0.75). All except one in the schizophrenia group received second-generation antipsychotic (SGA) medications (amisulpride, *n* = 6; aripiprazole, *n* = 13; clozapine, *n* = 8; olanzapine, *n* = 9; paliperidone, *n* = 1; quetiapine, *n* = 2; risperidone, *n* = 2), with the mean DDD of 14.83 mg of olanzapine equivalence/day (SD = 7.84). Schizophrenia patients showed lower estimated IQ (*p* < 0.001), immediate (*p* = 0.004) and delayed (*p* < 0.001) logical memory, LNT accuracy (*p* = 0.003) and longest category passed (*p* = 0.042) than controls, but the two groups had comparable immediate and delayed visual memory (*p*s > 0.05).

**Table 1:** Sample characteristics.

	Schizophrenia patients (*n* = 30)		Controls (*n* = 31)			
	Mean	SD	Mean	SD	*t*(_59)_/*χ*^*2*^	*p*
Age (years)	25.80	5.52	24.32	3.45	1.258	0.218
Gender (male vs female)^a^	15 vs 15		14 vs 17		0.143	0.705
Length of education (years)	13.67	2.60	16.129	1.78	−4.321	<0.001
Estimated IQ	95.79	8.48	108.10	10.56	−4.954	<0.001
LNT accuracy	15.43	3.82	19.16	5.40	−3.102	0.003
LNT longest category	5.97	1.35	6.74	1.55	−2.081	0.042
LM immediate recall	10.77	4.90	13.94	3.04	−3.043	0.004
LM delayed recall	8.33	4.37	12.32	3.50	−3.941	<0.001
VR immediate recall	20.97	2.44	21.03	2.65	−0.100	0.920
VR delayed recall	20.37	3.19	20.35	2.97	0.015	0.988
Defined Daily Doses (olanzapine equivalence, mg/d)	14.83	7.84				
DOI (years)	2.95	2.03				
DUP (years)	0.68	0.94				
PANSS positive subscale	8.03	2.43				
PANSS negative subscale	11.40	4.99				
PANSS general subscale	18.47	3.96				
HAMD	0.30	0.75				

*DDD* defined daily doses, *DOI* duration of illness, *DUP* duration of untreated psychosis, *HAMD* Hamilton Depression Rating Scale, *PANSS* the Positive and Negative Syndrome Scale, *LNT* Letter-Number Span test, *LM* logical memory, *VR* visual reproduction.

^a^χ^2^ test.

### Prospection performance

To adjust for multiple testing, only those *p*-values of <0.00454 could be regarded as reaching Bonferroni-adjusted significance. For participants’ subjective valence ratings, both the Group main effect (*F*[1,59] = 0.030, *p* = 0.864, ηp^2^ = 0.001) and the Group-by-Valence interaction (F[2,118] = 1.320, *p* = 0.271, ηp^2^ = 0.022) failed to reach statistical significance; however, the Valence main effect was significant even after Bonferroni adjustments (*F*[2,58] = 85.101, *p* < 0.001, ηp^2^ = 0.591) (see Table 2). Pairwise comparisons showed that all participants rated positive pictures more positively than neutral (*p* < 0.001) and negative (*p* < 0.001) pictures, but neutral and negative pictures were rated comparably (*p* = 0.649). For participants’ subjective arousal ratings, both the Group main effect (*F*[1,59] = 0.529, *p* = 0.470, ηp^2^ = 0.009) and the Group-by-Valence interaction (*F*[2,118] = 0.528, *p* = 0.588, ηp^2^ = 0.009) failed to reach statistical significance; however, the Valence main effect was significant even after Bonferroni adjustments (*F*[2,58] = 7.226, *p* = 0.001, ηp^2^ = 0.109) (see Table 2). Pairwise comparisons showed that all participants rated positive pictures (*p* = 0.002) and negative pictures (*p* = 0.001) as more arousing than neutral pictures, but the arousal ratings for positive pictures were comparable to negative pictures (*p* = 0.812).

**Table 2:** Affective prospective task results.

Schizophrenia patients (*n* = 30)							Controls (*n* = 31)						Group by valence repeated measure ANOVA results					
	Positive		Neutral		Negative		Positive		Neutral		Negative		Group main effect		Picture valence main effect		Group-by-picture valence interaction	
	Mean	SD	Mean	SD	Mean	SD	Mean	SD	Mean	SD	Mean	SD	*p*	ηp2	*p*	ηp^2^	*p*	ηp^2^
Subjective valence ratings	5.30	1.08	3.58	1.18	3.24	1.17	5.23	0.71	3.31	0.60	3.51	0.83	0.864	0.001	**<0.001**	0.591	0.271	0.022
Subjective arousal ratings	4.41	1.18	3.72	0.91	4.24	1.30	4.38	1.09	3.99	0.86	4.46	1.05	0.470	0.009	**0.001**	0.109	0.588	0.009
Pre-experience	4.51	1.18	4.61	1.07	4.10	0.89	4.91	0.88	4.82	0.76	4.23	1.06	0.222	0.025	**<0.001**	0.160	0.561	0.009
Temporal distance	3.16	1.10	2.48	1.16	3.12	1.06	3.25	1.21	2.63	1.01	3.00	1.17	0.846	0.001	**<0.001**	0.150	0.619	0.008
Emotion experience	5.09	0.88	4.17	0.81	3.28	1.09	5.17	0.78	3.63	0.76	2.97	1.03	0.086	0.049	**<0.001**	0.596	0.125	0.035
Vividness	4.67	1.09	4.60	0.96	4.28	0.93	4.99	0.75	4.63	0.82	4.34	0.90	0.475	0.009	**<0.001**	0.158	0.364	0.017
Participation	1.13	0.22	1.23	0.28	1.26	0.30	1.16	0.21	1.17	0.23	1.40	0.25	0.430	0.011	**<0.001**	0.161	0.034	0.056
Specificity^a^	2.46	0.74	2.35	0.63	2.60	0.59	2.92	0.19	2.81	0.35	2.95	0.12	**<0.001**	0.239	0.017	0.069	0.612	0.009
Richness of time/place^a^	1.47	0.53	1.21	0.72	1.18	0.72	1.55	0.54	1.73	0.71	1.42	0.58	0.039	0.073	0.038	0.056	0.036	0.056
Richness of sensory details^a^	0.99	0.86	0.73	0.65	1.09	0.84	1.27	0.73	1.15	0.72	1.33	0.70	0.077	0.054	**0.002**	0.100	0.461	0.013
Richness of thought/emotion^a^	0.83	1.10	0.93	1.03	0.95	0.96	1.87	0.69	1.79	0.69	1.92	0.72	**<0.001**	0.285	0.631	0.008	0.628	0.008

*P*-values at the Bonferroni-adjusted significance level of <0.00454 were bold.

^a^Data of 2 schizophrenia participants were excluded from analyses because of invalid prospective narratives.

Table 2 also shows the results for the self-report and coded data regarding prospection. For self-report phenomenal characteristics of the prospected events, the Group main effect for pre-experience (*F*[1,59] = 1.526, *p* = 0.222, ηp^2^ = 0.025), temporal distance (*F*[1,59] = 0.033, *p* = 0.856, ηp^2^ = 0.001), emotion experience (*F*[1,59] = 3.053, p = 0.086, ηp^2^ = 0.049), vividness (*F*[1,59] = 0.516, *p* = 0.475, ηp^2^ = 0.009), and participation (*F*[1,59] = 0.631, *p* = 0.430, ηp^2^ = 0.011) all failed to reach statistical significance.

The Valence main effect for pre-experience (*F*[2,58] = 11.247, *p* < 0.001, ηp^2^ = 0.160), temporal distance (*F*[2,58] = 10.438, *p* < 0.001, ηp^2^ = 0.150), emotion experience ([*F*2,58] = 86.983, *p* < 0.001, ηp^2^ = 0.596), vividness (*F*[2,58] = 11.043, *p* < 0.001, ηp^2^ = 0.158), and participation (*F*[2,58] = 11.286, *p* < 0.001, ηp^2^ = 0.161) all reached statistical significance after Bonferroni adjustments. Pairwise comparisons showed that all participants had greater sense of pre-experience in positive (*p* < 0.001) and neutral (*p* < 0.001) than negative prospection, perceived positive (*p* = 0.001) and negative (*p* = 0.001) prospection as farther in time than neutral prospection. All participants also experienced the highest pleasure in positive prospection, followed by neutral prospection and least in negative prospection (*p*s < 0.001). They perceived positive prospection as more vivid than negative prospection (*p* < 0.001) and neutral prospection (*p* = 0.05), and perceived neutral prospection as more vivid than negative prospection (*p* = 0.008). Moreover, the role of spectator was more likely to be adopted in negative prospection than in positive (*p* < 0.001) and neutral (*p* = 0.002) prospections.

The Group by Valence interaction effects for pre-experience (*F*[2,118] = 0.566, *p* = 0.561, ηp^2^ = 0.009), temporal distance (*F*[2,118] = 0.482, *p* = 0.619, ηp^2^ = 0.008), emotion experience (*F*[2,118] = 2.124, *p* = 0.125, ηp^2^ = 0.035), vividness (*F*[2,118] = 1.019, *p* = 0.364, ηp^2^ = 0.017), and participation (*F*[2,118] = 3.482, *p* = 0.034, ηp^2^ = 0.056) all failed to reach Bonferroni-adjusted statistical significance.

For the coded specificity and richness of participants’ prospected narratives, the Group main effect for specificity (*F*[1,57] = 17.903, *p* < 0.001, ηp^2^ = 0.239), and richness of thought/emotion (*F*[1,57] = 22.706, *p* < 0.001, ηp^2^ = 0.285) both reached statistical significance after Bonferroni adjustments, but not richness of time/place (*F*[1,57] = 4.467, *p* = 0.039, ηp^2^ = 0.073) and richness of sensory details (*F*[1,57] = 3.275, *p* = 0.077, ηp^2^ = 0.054).

The Valence main effect for richness of sensory details (*F*[2,56] = 6.366, *p* = 0.002, ηp^2^ = 0.100) reached statistical significance after Bonferroni adjustments, but not specificity (*F*[2,56] = 4.221, *p* = 0.017, ηp^2^ = 0.069), richness of time/place (*F*[2,56] = 3.372, *p* = 0.038, ηp^2^ = 0.056), and richness of thought/emotion (*F*[2,56] = 0.462, *p* = 0.631, ηp^2^ = 0.008). Pairwise comparisons showed, in all participants, negative prospected narratives (*p* < 0.001) and positive prospected narratives (*p* = 0.023) were richer in sensory details than neutral narratives.

The Group by Valence interaction effects for richness of time/place (*F*[2,114] = 3.408, *p* = 0.036, ηp^2^ = 0.056), specificity (*F*[2,114] = 0.494, *p* = 0.612, ηp^2^ = 0.009), richness of sensory details (*F*[2,114] = 0.779, *p* = 0.461, ηp^2^ = 0.031) and richness of thought/emotion (*F*[2,114] = 0.467, *p* = 0.628, ηp^2^ = 0.008) all failed to reach Bonferroni-adjusted statistical significance.

### Prospection performance after controlling for schizophrenia-associated memory deficits

When estimated IQ, LNT accuracy and longest category passed, immediate and delay logical memory were entered as covariates, the Group main effect for richness of thought/emotion (*F*[1,52] = 10.177, *p* = 0.002, ηp^2^ = 0.164) reached Bonferroni-adjusted statistical significance, but not specificity (*F*[1,52] = 7.738, *p* = 0.008, ηp^2^ = 0.130).

### Correlations of prospection performance and clinical variables

In schizophrenia participants, the specificity and richness of thought/emotion during (positive, neutral and negative) prospections were not associated with PANSS positive, negative and general symptoms (*p*s > 0.05). Moreover, the richness of thought/emotion of during positive, neutral and negative prospections were not correlated with illness duration and duration of untreated psychosis (*p*s > 0.05). However, the specificity of neutral prospection was significantly correlated with illness duration (*r*_s_ = −0.462, *p* = 0.013) and duration of untreated psychosis (*r*_s_ = −0.483, *p* = 0.009). The specificity of positive and negative prospections were not significantly correlated with illness duration and duration of untreated psychosis (*p*s > 0.05).

## Discussion

This study is one of the first few investigations on prospection in first-episode schizophrenia patients, and is an important extension of our earlier prospection research in chronic schizophrenia^15^. Both our previous^15^ and the current studies adopted the same task which utilized pictorial cues and collected self-report as well as transcribed and then coded data of the prospected events and narratives. According to the “constructive episodic simulation hypothesis”^17^, past memory is recombined and modified in prospection. Schizophrenia-associated memory deficits^18^ may confound prospection^3^. This study advanced further to employ covariate analysis to control for the effects of memory deficits on prospection in first-episode schizophrenia patients. In brief, our findings could be summarized as follows. First, first-episode schizophrenia patients self-report similar sense of pre-experience, temporal proximity, vividness and emotions during prospection as controls. Second, pictorial cues with varied emotion valence appeared to exert similar effects on these phenomenal characteristics of prospection to both first-episode schizophrenia patients and controls. This finding appeared to differ from our earlier findings^15^ that patients with chronic schizophrenia perceived closer temporal distance than controls when prospecting negative events. Third, first-episode schizophrenia patients’ prospected narratives were not as specific and rich in thought/emotion than controls, and the prospection deficits on the richness of thought and emotion persisted after controlling for schizophrenia-associated logical memory and working memory deficits. Taken together, our study extended empirical evidence for prospection deficits to first-episode schizophrenia patients.

Using the same prospection paradigm as in Yang et al.^15^, we found that first-episode schizophrenia patients showing largely intact phenomenal characteristics during prospection, whereas patients with chronic schizophrenia had diminished phenomenal characteristics in terms of lower sense of pre-experience and vividness of the prospected events^15^. However, first-episode schizophrenia patients appeared to exhibit more severe prospection deficits, as their prospected narratives were less specific and rich in thought/emotion than that of the controls, with effect size ranged from 0.239–0.285 (ηp^2^), which was greater than that found in our earlier chronic schizophrenia sample (ηp^2^ = 0.070, for richness of thought/emotion)^15^. Given that Yang et al.’s^15^ chronic schizophrenia sample had a comparable IQ to controls, but our two groups differed significantly in estimated IQ, covariate analyses were needed, which further suggested prospection deficits regarding richness of thought/emotion with effect size of 0.164 (ηp^2^).

Compared to other prospection research in the literature, our study adopted pictorial cues rather than sentences^3,13^, and therefore the findings were not directly comparable. Unlike previous studies^13,14^, our paradigm included neutral cues to examine emotion valence effects on prospection. Anticipatory pleasure deficits^8^ and failure in translating emotion into motivated behavior^19^ have been consistently found in schizophrenia patients. It is plausible that schizophrenia patients may differ from controls in prospecting events of varied valence. However, our findings and Yang et al.’s^15^ earlier findings both did not support this speculation. The small sample size of the current and previous prospection studies^3,13–15^ might have contributed to negative findings.

Prospection deficits in schizophrenia patients may contribute to negative symptoms and poorer functional outcomes^12^. Mental simulated representations/prospected events are important in predicting future pleasure and anticipatory affect^2^. According to the Kring and Barch’s^11^ temporal experience of emotion and reward-outcome translation model, anticipatory pleasure is an important component for negative symptoms in schizophrenia. Moreover, the accessibility model^20^ posits that ‘non-current’ defeatist beliefs and negative feelings are important in formation of negative symptoms, and therefore prospection may be one avenue to modify an individual’s non-current feeling. However, we did not find any correlation between prospection deficits and negative symptoms, likely attributable to our limited sample size.

In healthy people, prospection ability can be enhanced using “imagery cognitive bias modification”^21^ and the “guided episodic thinking”^22^. Imagery cognitive bias modification refers to a strategy to train people to habitually imagine positive resolutions of ambiguous information. For instance, Murphy et al.^21^ showed that the prospected events became more vivid, after imagery cognitive bias modification; and healthy people with more vivid prospection had less negative affect/ trait anxiety and more optimism. On the other hand, guided episodic thinking refers to a protocol-based, interviewer-guided strategy to increase the vividness and details of mental imagery of past and future events. For instance, Hallford et al.^22^ found guided episodic thinking improved the vividness of prospection; and healthy people with improved prospection had more anticipatory pleasure and behavioral intention. These strategies^21,22^ have not yet been examined in schizophrenia patients. However, the relationship between positive prospection and depressive symptoms have been examined^23,24^, and patients with major depressive disorder (MDD) who had more vivid positive prospection were found to be more optimistic^23^. Renner et al.^25^ also reported that positive prospection could bring better behavioral activation in MDD patients. Future research should investigate these intervention strategies^21,22^ on prospection deficits in schizophrenia patients, and whether it would eventually improve negative symptoms and functional outcome.

This study has several limitations. First, our sample size was modest, which might have resulted in negative findings regarding differential effects of valence of cues on schizophrenia patients’ prospection. Second, our schizophrenia and control groups were not matched in education and estimated IQ. Having said that, we have employed covariate analysis to control for difference in IQ and cognitive functions. Third, we only measured non-verbal IQ, but the ability to generate narratives may be related to verbal-IQ and language production ability. For instance, schizophrenia is associated with language production deficits at various levels (such as words, sentences, narratives)^26^; verbal-IQ also covers abstract verbal reasoning, verbal working memory, and lexical knowledge. We did not control for these functions which might have confounded our findings. Lastly, our behavioral study did not examine putative neural correlates for prospection deficits in schizophrenia patients, though altered functional connectivity between the retrosplenial cortex and the insular, and altered functional connectivity between the hippocampus and the parahippocampus have been reported as neural substrate for affective forecasting in people with social anhedonia^2^.

Notwithstanding these limitations, our findings have implications on future research and clinical practice. Anticipatory pleasure deficits could emerge early in the course of schizophrenia; and our preliminary findings support the existence of prospection deficits in first-episode schizophrenia patients. Difficulty in simulating and pre-experiencing future events may constitute clinical problems, affecting vocational training and cognitive remediation. Our preliminary findings support further research on the potential benefits of prospection-enhancing strategies^21,22^ to first-episode schizophrenia patients. Early identification and intervention to prospection deficits may improve functional outcome.

## Conclusions

To conclude, first-episode schizophrenia patients exhibit prospection deficits. Future study should utilize a large sample to clarify our preliminary findings, and explore the relationship of prospection deficits with negative symptoms and functional outcome.

## Methods

### Participants

We recruited outpatients with first-episode schizophrenia from an early psychosis intervention programme at Castle Peak Hospital Hong Kong from August 2020 to May 2021. Moreover, 31 healthy individuals were recruited from neighboring community as controls. Eligibility criteria included (1) ethnic Chinese, (2) estimated IQ > 70, (3) normal or corrected hearing or vision, (4) absence of any history of head injury with loss of consciousness for >30 min, (5) absence of any neurological disorders, and (6) no history of substance abuse nor electroconvulsive therapy (ECT) in the last 6 months. Moreover, all controls did not have any Axis I DSM-IV psychiatric disorder, nor family history of psychotic disorder. The clinical diagnosis was ascertained using the structured clinical interview of SCID-I^27^, supplemented by medical records. We gathered clinical participants’ demographics, and clinical characteristics, and estimated the defined daily doses (DDDs)^28^ of antipsychotic medications in terms of olanzapine equivalence.

### Affective prospection task

The validated computer-based Affective Prospection Task^15^ was used. In this task, participants viewed 9 pictures (3 positive, 3 neutral, and 3 negative) selected from the International Affective Picture System (IAPS^29^) and the Chinese Affective Picture System (CAPS^30^). The positive pictures were (1) colorful flowers and trees and several people in a garden (IAPS cue code 5199), (2) a beach scene with two people looking at the sea (IAPS cue code 5836), and (3) food and drinks on a table (CAPS cue code 125). The neutral pictures were (1) an ironing board with clothes (IAPS cue code 7234), (2) two Asian men resting on the subway (IAPS cue code 2397), and (3) standing on a weighing scale (IAPS cue code 7044). The negative pictures were (1) a person drawing blood from another person (IAPS cue code 9592), (2) flies on a pie (IAPS cue code 7360), and (3) garbage and a working man (IAPS cue code 9342). Before the formal task, participants underwent practice trials to ensure their understandings of task instructions. In each trial of the formal task, participants viewed one picture, and indicated their subjective valence (1 = very unpleasant, 7 = very pleasant) and arousal ratings (1 = very low arousal, 7 = very high arousal) on a 7-point Likert scale. They then generated a narrative by ‘prospecting an event’ which may happen to them. They were instructed that all prospected events should be new, rather than mere recollections of past events. Moreover, the prospected events should be specific in ‘time’ and ‘place’, lasting not longer than one day. The prospected events could include contents of the pictures they viewed. The picture was presented continuously on the screen while participants were prospecting. Participants then made a prospected narrative with as many as details as possible, including (1) the time and place of the event, (2) sensory details, and (3) thoughts and emotions. Participants’ prospected narratives were recorded and transcribed for coding by trained raters.

In this study, we found 33 invalid/missing trials (out of 270 trials) in the schizophrenia group for reason of failure to generate any prospected events. Moreover, 2 schizophrenia participants’ failed to provide any valid prospected narrative. On the other hand, no invalid/missing trial was found in the control group. After reporting their prospected narratives, participants rated the phenomenal characteristics of the prospected events and the current experience while prospecting the events^15^. Specifically, they rated on (1) sense of pre-experiencing (1 = not at all, 7 = completely), (2) temporal distance (1 = will happen in less than 1 week, 7 = will happen more than 5 years later), (3) emotion experience during prospection (1 = very unpleasant, 7 = very pleasant), (4) vividness (1 = no image at all, 7 = very clear and vivid) and (5) levels of participation (1 = a participant, 2 = a spectator) on a 7-point Likert scale. We counterbalanced the presentation of three sets of pictures with different valence, and randomly presented the 3 pictures from the same set.

### Other measurements

Participants’ non-verbal IQ was estimated using the TONI-4^31,32^. Moreover, we administered the logical memory subscale and visual memory subscale of the Wechsler Memory^33^, and the Letter Number Span test (LNT)^34^. In the LNT, participants were presented with a series of alternating letters and numbers, and were asked to rearrange the letters and numbers in successive order. We calculated the LNT accuracy (ranged 0 to 32) and LNT category passed (ranged 2 to 9). Higher LNT scores indicate better working memory. In addition, psychopathology in clinical participants was measured using the Positive and Negative Syndrome Scale (PANSS^35^) and the Hamilton Depression Rating Scale (HAM-D^36^).

### Data analysis

Data of participants’ narratives regarding their prospected events was transcribed for coding by two independent trained raters blind to the group membership and hypothesis of the study^15^. For each prospected narrative, the raters coded its specificity and richness of the narrative. For specificity, a coding of 3 refers to specific events that occurred at a specific place and time (i.e., high specificity), which lasted less than 1 day; a coding of 2 refers extended events that lasted more than 1 day; and a coding of 1 refers repeated events representing a category of events that might happen repeatedly over a long time (i.e., low specificity); a coding of 0 means participants could not generate an prospected events (i.e., missing data). Higher scores indicated greater specificity. For richness, the raters covered three domains: (1) time/place, (2) sensory aspects of the event, and (3) thought/emotion details. Richness of detail in these three domains was assessed on a 3-point scale with higher scores representing higher levels of richness. For example, descriptions that were rich, vivid, and highly specific and seemed to arise from a sense of pre-experiencing would be rated 3. Descriptions that were less rich were rated 2, and descriptions that were limited to general, nonspecific information but were still episodic in nature would be rated 1^15^.

All statistical analyses in this study were conducted using SPSS version 26. Demographics, estimated IQ, and memory functions of schizophrenia participants were compared with that of controls using χ^2^ test and independent sample t-tests. Participants’ valence and arousal ratings on the 9 pictorial cues were entered into 2 repeated measure ANOVAs, with Group as the between-subject factor, and Picture Valence as the within-subject factor. To compare the group difference in self-report pre-experience, temporal distance, emotional experience, vividness and participation in the prospected events, we conducted a series of repeated measure ANOVAs, with Group as the between-subject factor, and Picture Valence as the within-subject factor. Moreover, to compare the group difference in specificity, richness of time/place, richness of sensory details and richness of thought/emotion of the prospected narratives, we conducted a series of repeated measure ANOVAs, with Group as the between-subject factor, and Picture Valence as the within-subject factor. Bonferroni adjustments were employed to correct for multiple testing of group differences in prospection. In addition, covariate analyses were used to control for the effects of estimated IQ and memory deficits on prospection ability. Lastly, we explored the relationship of schizophrenia-associated prospection deficits with the PANSS subscales, illness duration and duration of untreated psychosis in the schizophrenia subgroup using Spearman’s correlations. Given that the correlation analysis was explorative, we did not apply Bonferroni adjustments.

### Procedures

We first administered the PANSS, SANS, HAM-D, and then TONI-4 and the neurocognitive battery, and lastly the Affective Prospection Task. The assessments were conducted within the same day in a quiet room, with breaks between the tasks to avoid participants becoming fatigued. This study was approved by the Ethics Committee of the New Territories West Cluster, Hospital Authority Hong Kong (Protocol number: NTWC/REC/20083). All participants provided written informed consent.
